# Biomarker responses in *Danio rerio* following an acute exposure (96 h) to e-waste leachate

**DOI:** 10.1007/s10646-024-02784-6

**Published:** 2024-07-12

**Authors:** A. Rielly, S. Dahms-Verster, R. Greenfield

**Affiliations:** 1 Department of Zoology, University of Johannesburg, Auckland Park, Johannesburg, South Africa; 2 School of Geography, Archaeology and Environmental Studies, Wits University, Johannesburg, South Africa

**Keywords:** Biochemical responses, Biomarkers, e-waste, Freshwater, Metals, Physiological effect

## Abstract

Electronic waste (e-waste) has been identified as an emerging pollutant and is the fastest growing waste stream at the present time. Significant technological development and modernization within the last decade has led to the rapid accumulation of outdated, broken and unwanted electrical and electronic equipment (EEE). Electronic products mainly consist of a range of metal containing components that, when disposed of improperly, could result in metal constituents leached into the environment and posing a health risk to humans and animals alike. Metal exposure can induce oxidative stress in organisms, which could lead to synergistic, antagonistic and additive effects. The metals found highest in abundance in the simulated e-waste leachate, were nickel (Ni), barium (Ba), zinc (Zn), lithium (Li), iron (Fe), aluminium (Al) and copper (Cu). An acute exposure study was conducted over a 96 h period to determine the potential toxicity of e-waste on the test organism *Danio rerio*. Biomarker analysis results to assess the biochemical and physiological effects induced by e-waste leachate, showed a statistically significant effect induced on acetylcholinesterase activity, superoxide dismutase, catalase activity, reduced glutathione content, glutathione s-transferase, malondialdehyde and glucose energy available. The Integrated Biomarker Response (IBRv2) analysis revealed a greater biomarker response induced as the exposure concentration of e-waste leachate increased.

## Introduction

Significant technological developments and modernization of the last decade, led to the emergence of a new pollutant, referred to as electronic waste (e-waste) (Shaikh et al. 2020; Soetrisno and Delgado-Saborit 2020). E-waste has been identified as the fastest growing waste stream due to the abundance of electrical and electronic equipment (EEE) that has been engrossed and integrated in everyday life. The decline in lifespan of electronics and new technology has also subsequently been observed—presumably as a ploy to encourage consumers to regularly replace and upgrade to the new and improved (Kolias et al. 2014). Consumer knowledge and/or consumer accessibility to proper disposal of electronics adds to this pollutants’ accumulation (Singh et al. 2020).

As such, e-waste has formally been defined as any electrical and electronic device or product discarded because it is deemed as obsolete, unappealing, or broken by its consumer (Zeng et al. 2020). Some recent publications estimate the global production of e-waste, to have reached 52.2 million tons produced in 2021 alone, from which only 20–25% was formally recycled (Li and Achal 2020; Shaikh et al. 2020). The unaccounted e-waste is presumed to either be stored in household homes or located at municipal dumps where they are informally recycled by low-income workers (Li and Achal 2020; Shaikh et al. 2020). The known rudimentary processes for the breakdown of e-waste, used by informal recyclers, involve open dumping, open burning, manual dismantling and shredding.

These recycling techniques are considered responsible for exposing a wide range of contaminants that form part of electronics, into the surrounding ambient environments. E-waste contains more than 1000 different substances overall, which can be classified as either hazardous or non-hazardous (Kolias et al. 2014; Rao et al. 2017). The most prominent of these substances include metals, persistent organic pollutants (POPs) and brominated flame retardants (BFR) (Boa et al. 2020; Li and Achal 2020; Zeng et al. 2020). The exact composition of e-waste, however, is dependent on factors such as what type of electronic device it is, the model of the device, the manufacturer, date of manufacturing, as well as the age of the electronic device. Metals constituents reportedly accounts for roughly 60% of the hazardous compounds typically found in e-waste, as summarised in Table 1 (Mmereki et al. 2016; Rao et al. 2017).

**Table 1:** List of common metal elements that are typical constituents of electronic wastes and their environmental and health relevance

Substance	Occurrence in electronic waste	Environmental and health relevance.
Lead (Pb)	Used as shielding in cathode ray tubes (CRTs) and solder for circuit boards (PCBs).	Influence the nervous system, kidneys, blood-forming organs, reproductive systems and cardiovascular system of foetus, children and women of childbearing age.
Cadmium (Cd)	Used in semiconductors and solder.	Class I carcinogen and produces toxicity in kidneys, skeleton, reproductive organs and affects all age groups, but females and the elderly seem at a special risk for adverse outcomes.
Arsenic (As)	Found in small quantities in the form of gallium arsenide within light-emitting diodes (LEDs).	Acutely poisonous by inhibiting cellular respiration and increasing apoptosis and oxidative stress.
Mercury (Hg)	Used in flat panel screens such as televisions and liquid-crystal displays (LCDs), and on/off switches.	Affects at a subcellular level of organisation and leads to cell deaths.
Gallium (Ga)	Commonly used on computer chips, cellular devices and LEDs.	Interferes with cell-cycle division processes and induces a stress protein response.
Indium (In)	Used in a variety of high-speed electronics, such as computers, mobile phones and flat-panel screens.	Highly toxic and capable of inhibiting protein synthesis, exacerbating lung diseases and is carcinogenic.
Barium (Ba)	Used in CRTs.	Has gastrointestinal effects and may lead to cardiovascular, neurological, or neuromuscular weaknesses.
Tin (Sn)	Used to strengthen and harden alloying elements on the PCBs solder and LCDs.	Induces psychological and developmental implications. Liver damage and sub-cellular alterations.
Antimony (Sb)	Present in tin-lead alloys, in printed circuit boards (PCBs) and is used to harden alloys further.	Acutely poisonous by inhibiting cellular respiration and increasing apoptosis and oxidative stress. May also lead to cardiovascular and respiratory problems.
Nickel (Ni)	Predominately found in batteries but is also used in printed circuit boards (PCBs) in small amounts.	In high concentrations it becomes toxic and may alter metabolic activities by inhibiting enzymes. It may also act as an immunotoxin and carcinogenic agent.
Chromium (Cr)	Used as an allotting element to prevent corrosion of steel. It is present in PCBs and floppy disks.	The toxicity depends on the valence of Cr. In its toxic forms, it results in increased stress proteins. It also has a mutagenic and carcinogenic effect at high concentrations and could cause kidney necrosis etc.
Copper (Cu)	Used because of its conductive properties in PCBs and cables.	High exposure can lead to the accumulation of Cu in the body and then results in oxidative damage, neurodegenerative diseases, and metabolism fatigue.
Zinc (Zn)	Used in manufacturing or PCBs and LCDs.	Persistence in the environment and tends to accumulate in the body. With excessive exposure it leads to pancreatic damage, disturbance in protein metabolism as well as respiratory disorders.

Table adapted from Pant et al. (2012); Fowler (2017); Iqbal et al. (2019) and Singh et al. (2020)

The electronic waste that falls under the ‘IT and telecommunication’ e-waste classification has a higher presence of precious and valuable metals than any other category of e-waste according to Mmereki et al. (2016). The purpose of this study, therefore, is to assess how the emergence of this new pollutant which has the potential to cause major environmental destruction, would affect biological organisms even in trace amounts. Organisms in the natural environment are always exposed to numerous different contaminants with different modes of action, and each contaminant will contribute to the final overall adverse resulting effect (Boa et al. 2020). Different components that form part of e-waste, in different e-waste mixtures will subsequently release different concentrations of metals, which can then interact and produce a synergistic, antagonistic or additive effect on the exposed organism, and potentially lead to altered physiological toxicities (Boa et al. 2020; Rodrigues et al. 2020).

Biomonitoring of ecosystems or pollution exposures is aimed at screening, identifying, and understanding how anthropogenic activities affect the condition or health of environments and biological organisms (van der Oost et al. 2003; Costa and Teixeira 2014). These assessments are completed by utilising bioindicator species, to provide information about cause-and-effect relationships between the environment and the induced stressor. The test organism chosen for this study, *Danio rerio* (common name: Zebrafish) is one of the most used model organisms in biological research studies, worldwide (Khan and Alhewairini 2018). *Danio rerio* are tropical freshwater fish that have many attributes which make them an ideal bioindicator species—such as being small, hardy, inexpensive and easy to house (Spence et al. 2008). The aim of this assessment was to (i) determine the broad array of metal toxins associated with leached e-waste Waste Printed Circuit Boards (WPCBs), and (ii) to assess the combined biochemical and physiological stress effects e-waste leachate induce on bioindicator species, *Danio rerio*, following an acute exposure (96 h) to simulated e-waste leachate. These effects are to be assessed through the use of biomarkers of exposure and biomarker of effects. The suite of biomarkers selected to evaluate the biological responses included acetylcholinesterase activity (AChE) and metallothionein (MT) for exposure; catalase activity (CAT), reduced glutathione (GSH) content, glutathione S-transferase (GST) activity, malondialdehyde (MDA) content, protein carbonyl (PC) and superoxide dismutase (SOD) for effect; and cellular energy activity (CEA) for energetics. We hypothesised an acute leachate exposure on *Danio rerio* will result in a range of measurable biochemical and physiological responses, and that an increase in exposure concentrations will positively correlate to an increase in measurable biomarker responses.

## Materials and methods

### Electronic waste dismantling

Printed circuit boards (PCBs) of discarded computers, from different brands, and random-access memory (RAMs) of waste computers were collected from the University of Johannesburg’ Doornfontein Campus. After collection, the PCBs and RAMs were carefully cleaned of dust, and manually dismantled from their external components. They were then further dismantled, by being cut into smaller pieces to ensure increased surface area contact with the leaching medium. According to Meem et al. (2020), the increased surface area increases reactivity and speeds of the chemical reactions. Consumables used for manual dismantling and to ensure safety from potential exposure to metals includes protective eyewear, polyester protective latex gloves and tools such as a 250 mm tin snip shears, groove joint pliers, side-cutter, long-nose pliers, flat screwdriver, and a ratchet screwdriver with interchangeable tips.

### Exposure leaching

The leaching approach to collect simulated leachate for an acute exposure study on *D. rerio*, was a dynamic non-acidic leaching process, where an 800 L glass tank was filled with borehole water in a 1:10 (Solid: Liquid) ratio. The exposure leaching was done for a duration of 4 weeks. In-situ water parameters were recorded, every day, at the same time and water samples were collected for metal distribution and concentration analysis at the end of the leaching cycle. The leaching procedure was completed in a controlled environmental room, with the room temperature set to 28 °C. Following the duration of leaching, the leachate solution was stored in a glass tank and sealed to limit leachate loss via evaporation.

### Exposure design

All exposures were conducted in controlled environmental rooms, to maintain a constant temperature and light-dark cycle, as well as to limit the influence of unforeseen events. The acute exposure was conducted over a 96 h period, in duplicate. Eighty (n = 80) *D. rerio* fish were separated into four 100 L glass aquarium tanks, with twenty fish (n = 20) per tank. Ten (n = 10) fish from the twenty fish in each tank, are dedicated to biomarker analyses, and the remaining ten (n = 10) *D. rerio* are dedicated for both bioaccumulation and biomarker analysis.

### Acute Exposure

The *D. rerio* fish were left to acclimatise in oxygenated borehole water for a period of two weeks, at temperatures ranging between 25 °C and 28 °C and a light-dark cycle of 14/10 h. Upon the start of the exposure, e-waste leachate stock solution—obtained from the leaching procedure preceding the acute exposure—was added to each tank to make up the exposure concentrations which consisted of a control (0% leachate), 25% of leachate stock, 50% stock solution and 100% of the stock solution. Electronic waste leachate was filtered through a 53 micromolar (µm) Endecott, stainless steel, wet-washing laboratory test sieve (ISO 3310-1) prior to being added to exposure tanks. The exposure was conducted over a 96 h period with a 10% water change done daily. Water samples were also collected daily, before and after each water change, for water quality testing.

### Biomarker analysis and tissue processing

Viscera and muscle tissue samples were collected, weighed, and placed into prelabelled microcentrifuge tubes and homogenised in the appropriate buffer (related to the protocols below) using a CAT Scientific X120 homogeniser. Samples were kept cold on ice, as to prevent enzymatic degradation. Viscera tissue samples were used for all enzymatic biomarkers, whereas muscle tissue was used for the determination of energetic biomarkers. Colorimetric determination was done by using 96-well microtiter plates and a Synergy HTX multimode reader. All samples were read in triplicate to validate the empirical data. Biomarkers were standardised by measuring the protein content of each solution used.

Acetylcholinesterase (AChE) activity was determined according to Ellman et al. (1961) and Xuereb et al. (2009); by incubating 20 µL of sample supernatant, 260 µL 0.1 M Potassium Phosphate Buffer and 20 µL 0.01 M Ellman’s (2,2-Dinitro-5,5′5dithio-dibenzoic acid) reagent for 5 min at 25 °C. Following incubation, 10 µL of 0.075 M s-acetylthiocholine iodide was added to each well in the microtiter plate. The reaction was measured at 412 nm at 1 min intervals, over a period of 6 min.

The metallothionein (MT) content was measured according to Viarengo et al. (1997); whereby samples were centrifuged at 6000 × *g* for 10 min at 4 °C, after 250 µL of sample supernatant, 265.5 µL cold absolute ethanol (−20 °C) and 20 µL chloroform were added to a new sterilised microcentrifuge tube and vortexed for 15 s. Supernatant formed was transferred to new microcentrifuge tubes and pellet was discarded. An addition of 1.5 mL acidified ethanol was added to each sample and incubated for 1–2 h at −20 °C and centrifuged for 10 min, at 6000 × *g* and 4 °C. Sample supernatant was discarded, and pellet formed washed twice with 1 mL buffer; The pellet was dried and resuspended in 37.5 µL 0.25 M NaCl and 37.5 µL HCl-EDTA and vortexed. Procedural blanks (15 µL MT homogenising buffer), 15 µL sample and 210 µL DTNB were added to 96-well microtiter plates and read at an absorbance of 412 nm.

Superoxide dismutase (SOD) activity was determined following the method of Del Maestro and McDonald (1989); whereby 4 µL sample supernatant and procedural blanks, with 242 µL Tris-HCl-DTPA and 4 µL of 24 mM Pyrogallol were added to the microtiter plate wells for analysis. The plates were analysed kinetically over a 4 min period with 30 s intervals at 420 nm.

Catalase activity (CAT) followed the method of Cohen et al. (1970), beginning with 10 µL of sample and blanks added to microtiter plate wells, as well as 100 µL 6 mM H_2_O_2_ and 10 mM KP-buffer. The microtiter plates were incubated for 3 min at room temperature (23 °C) and an additional 20 µL 6 N H_2_SO_4_ was added thereafter, to stop the reaction. After the reaction ceased, 140 µL of 0.01 N KMnO_4_ was added, and the plate measured at 480 nm.

Malondialdehyde (MDA) activity was determined following the method of Ohkawa et al. (1979) and modified by Üner et al. (2006). This was assessed by centrifuging samples at 10,000 × *g* for 10 min at 4 °C. From which, 40 µL of sample supernatant, 40 µL SDS, 375 µL 20% acetic acid, 375 µL 0.8% thiobarbituric acid and 175 µL deionised water were thoroughly vortexed together and incubated at 95 °C for 60 min. Samples were allowed to cool down to room temperature. Once cooled, 200 µL deionised water and 1000 µL butanol-pyridine solute was added and centrifuged at 900 × *g* for 10 min. From this, 245 µL sample and 245 µL procedural blank (tris-sucrose buffer) were added to microtiter plates and measured at 532 nm.

Protein carbonyl (PC) concentrations, were measured according to Floor and Wetzel 1998) and Levine et al. (1990); where 100 µL sample supernatant and 500 µL 10 mM DPNH were mixed with 2 M HCL, incubated at room temperature for 60 min, and vortexed every 10–15 min. An equal amount of 6% TCA was added, vortexed, and then centrifuged at 11,000 × *g* for 3 min at 23 °C. The resulting supernatant was discarded, and pellet formed was washed with 1 mL ethanol-ethyl acetate and centrifuged at 11,000 × *g* for 3 min, three times. The pellet was dissolved in 400 µL 50% formic acid and 6 M guanidine hydrochloride, while centrifuged at 16,000 × *g* for 5 min. Thereafter, 100 µL of sample supernatant was added to 96-well microtiter plates and measured at 360 nm.

Reduced glutathione (GSH) was determined following the method by Cohn and Lyle (1966); where a 500 µL tissue sample and 15 µL H_3_PO_4_ were centrifuged at 5000 × *g* for 10 min at 4 °C, after which 10 µL of the supernatant was mixed with 1000 µL deionised water. From this, 6 µL of sample and 6 µL GHB (blank) were added to each microtiter plate, as well as 232 µL sodium phosphate buffer and 12 µL OPT to each microtiter well. After an incubation period of 15–20 min at 23 °C, samples fluorescence was read at 420 nm.

Glutathione S-transferase (GST) activity was analysed according to Habig et al. (1974) and Javed et al. (2016); where a volume of 10 µL of sample and blanks were added to microtiter plate for analysis, along with 270 µL GSH-reaction mixture (0.1 M KP-buffer (pH 6.5) + 1 mM GSH) and 20 µL 1 mM CDNB-ethanol. Samples were read immediately and measured kinetically at 340 nm, every 30 s for a period of 3 min.

Cellular energy allocation (CEA) was determined by following the method of De Coen and Janssen (2003); where energy consumption samples were centrifuged at 3000 × *g* for 10 min at 4 °C. From the supernatant, 25 µL and 25 µL of the ETS homogenising buffer (blank) were added to each well of a 96-well microtiter plate. Cold buffered substrate solution (75 µL), along with 25 µL NAD(P)H solution and 50 µL INT were added to each well before being kinetically analysed at 490 nm. Energy reserves were established by determining glucose, lipid and protein content within muscle tissue of the fish. Glucose availability was determined by the method outlined by Masuko et al. (2005). The assay involved using 50 µL of blank and sample supernatant added to microtiter plates and mixed with 150 µL H_2_SO_4_ and 30 µL 5% Phenol. The plates were incubated for 5 min at 90 °C and left to cool down to room temperature before being analysed at 490 nm. Protein availability was determined using the methodology of Bradford (1976) and lipid availability by following the procedure outlined by Bligh and Dyer (1959); where 500 µL of sample supernatant and 500 µL of chloroform were added to sterile microcentrifuge tubes and vortex thoroughly. Thereafter, 500 µL methanol and 200 µL deionised water was added and the mixture was centrifuged at 3000 × *g* at 4 °C for 5 min. The organic phase (100 µL) was transferred to glass test tubes and 500 µL 98% H_2_SO_4_ added, capped with tinfoil and incubated at 200 °C for 15 min. After incubation, 1000 µL deionised water was added to each test tube, thereafter, 245 µL of sample and procedural blanks were added to polyethylene microtiter plates and analysed at 405 nm. Cellular energy allocation was calculated as CEA = Ea − Ec, where Ea was calculated as the sum of glucose, lipids and proteins.

### Statistical analysis

Parametric assumptions for normality were assessed using a Shapiro–Wilk test. Data sets that violated the assumption for normality, were first square root transformed and retested—if the data set still did not conform, it was natural log transformed and tested again (Laerd Statistics 2017). Thereafter, if the assumption for normally distributed data was not met, non-parametric tests were applied instead. A Levene’s test was used to assess the homogeneity of variance of data sets that conformed to the assumption of normality. Statistically significant differences (p < 0.05) for data sets that conformed to both parametric assumptions were determined by using a one-way analysis of variance (ANOVA) and further investigated with a Tukey Post Hoc test to identify differences between groups (Field 2009; Laerd Statistics 2017). If the assumption for homogeneity of variance was violated, a robust Welch ANOVA and a Games–Howell Post Hoc test was run (Laerd Statistics 2017). Non-parametric test ran on the data sets that violated the parametric assumptions, included a Kruskal–Wallis H test and a Dunn–Bonferroni Post Hoc test to determine significant differences (Field 2009; Dinno 2015; Laerd Statistics 2017).

Twenty-six individual metal elements were tested for in e-waste leachate: Al, Sb, As, Ba, Be, Bi, Cd, Cr, Co, Cu, Ga, Ge, Au, In, Fe, Pb, Li, Hg, Mo, Ni, Se, Ag, Sn, V, Y, and Zn.

Statistical analysis was completed by utilising a statistical package for social sciences (SPSS) version 28 (International Business Machines Corporation (IBM) Software Group). Graphical illustrations representing the individual elements leached during the test leaching process were done using GraphPad Prism (Prism 5 for Windows, version 5.02).

The IBRv2 response was calculated for each concentration for the acute leachate exposure study on *D. rerio*. In order to calculate the IBRv2 score, individual biomarker results were log-transformed to reduce variance and compared to reference data (control group (A1)). To calculate the mean standardised biomarker responses, transformed data was standardised according to the general mean and SDs of the data set. Biomarker deviation indices were calculated by subtracting the mean reference biomarker data from the mean standardised biomarker responses for each concentration. The IBR was then calculated as the sum of the absolute values of the biomarker deviation indexes (Sanchez et al. 2013).

## Results

Leachate elemental concentrations and biomarker data are all reported in text as the ANOVA F statistic, Welch’s F statistic or Kruskal–Wallis H statistic. The degrees of freedom for the effect of exposure, the degrees of freedom for residuals of the model, the significance (p) relative to the model, as well as the effect sizes (ω^2^ and ε^2^), are reported. Alpha thresholds were set to 0.05, but it was also reported if the significance was less than 0.01. Effect sizes that were determined as negatives are indicative of F statistic being <1.

### Physico-chemical parameters and elements detected

**Fig. 1:**
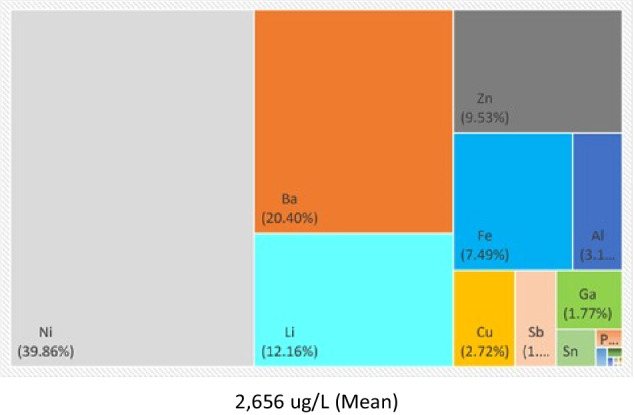
Graphical representation of elemental concentration at the conclusion of the final leaching cycle

Physico-chemical parameters measured during the final leaching procedure are summarised in Table 2; where the temperature and pH fluctuations during the final test leaching procedure was stable as depicted in. A full detection range for metals leached from WPCBs is presented in Table 3 with mean ± SD displayed. Twenty-five metals were tested for, from which only 19 were within detectable range for the leachate. A graphical representation of the tank and the metals highest in concentration is presented as percentages, in Fig. 1; which indicates the metal distribution with Ni > Ba > Li > Zn > Fe > Al > Cu as the most abundant metals in concentration.

**Table 2:** Overall mean physico-chemical parameters measured for the final leaching procedure

Tank	Temp. (°C) ± SD	pH ± SD	CD (µS/cm) ± SD
Final leaching	28.9 ± 0.037	7.54 ± 0.247	389 ± 37.0

**Table 3:** Mean ± standard deviation of elements detected for the final leaching cycle

Elements	Mean (ug/L)	SD	Elements	Mean (ug/L)	SD
Ag	0.77	0.439	Ge	0.279	0.045
Al	82.5	27.6	Li	323.0	86.1
As	0.355	0.095	Mo	1.74	0.253
Au	0.137	0.154	Ni	1059.05	362.0
Ba	542.0	42.7	Pb	5.95	3.01
Co	2.70	0.569	Sb	48.3	3.54
Cr	0.193	0.308	Sn	18.2	6.63
Cu	72.4	14.3	Y	0.053	0.022
Fe	199.0	84.9	Zn	253.0	35.8
Ga	47.1	8.42

The physico-chemical parameters measured during the acute exposure study (Table 4) were all within the required targeted ranges for the test organisms (Ross 2004; Avdesh et al. 2012). Temperature and pH values remained stable across all exposure groups with an average SD of 0.204 and 0.225, respectively. The EC decreased, across exposure groups, throughout the exposure period. The average EC decreased as the e-waste concentration increased; the two highest concentrations (50 and 100%) exhibited the lowest EC.

**Table 4:** Physico-chemical parameters measured for exposure water, during *Danio rerio* exposure to three concentrations of electronic waste leachate over 96-hrs

Group	Temp. (°C) ± SD	pH ± SD	CD (µS/cm) ± SD
A1 (Control)	23.8 ± 0.295	7.57 ± 0.374	1063 ± 89.8
A2 (25% leachate)	23.7 ± 0.194	7.67 ± 0.247	842.1 ± 84.4
A3 (50% leachate)	23.8 ± 0.195	7.80 ± 0.156	754 ± 53.7
A4 (100% leachate)	23.9 ± 0.133	7.91 ± 0.124	418 ± 11.5

### Biomarkers of exposure and effect

**Fig. 2:**
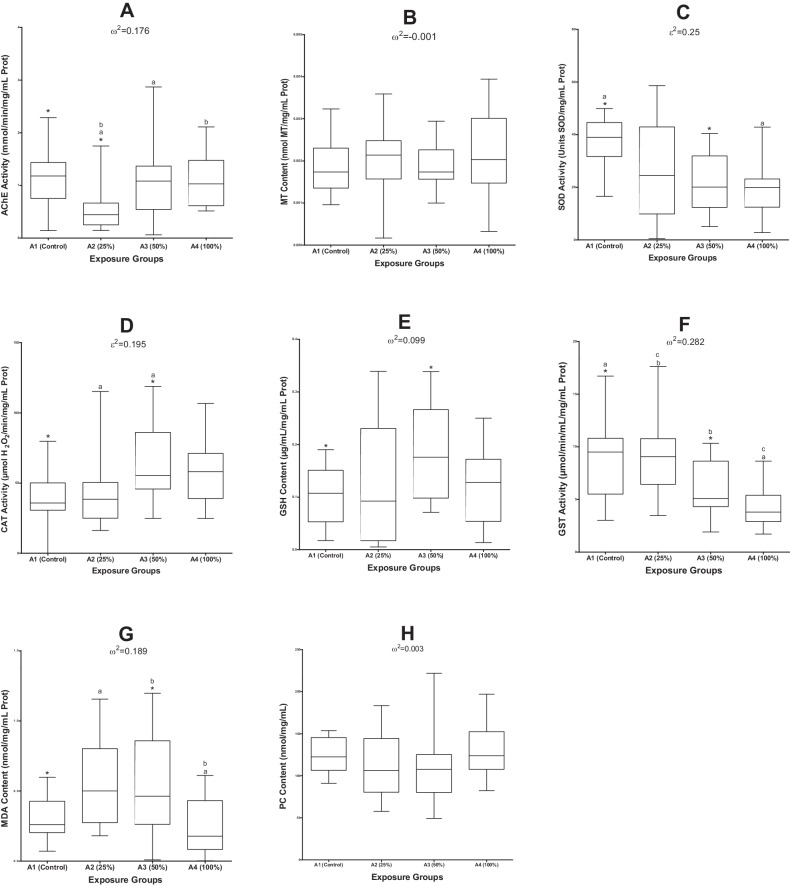
Box-and-whisker plots that show the 5th to 95th percentile range of (**A**)—AChE, (**B**)—MT, (**C**)—SOD, (**D**)—CAT, (**E**)—GSH, (**F**)—GST, (**G**)—MDA, (**H**)—PC. Common superscripts indicate statistically significant differences (p < 0.05) between exposure groups. Omega squared (ω^2^) and epsilon squared (ε^2^) show effect sizes

Biomarkers of exposure and effect are represented in Fig. 2; where *Danio rerio* were classified into four groups for the acute e-waste exposure study, namely as the control group (A1), the 25% leachate exposure group (A2), 50% exposure group (A3), and the 100% leachate exposure group (A4).

Acetylcholinesterase activity (Fig. 2A) showed an effect size, ω^2^ = 0.176, which signifies a large effect. A decrease in mean AChE activity was observed, from A1 to A4, A3 and A2, in that order. A Games–Howell Post Hoc test indicated a significant decrease in AChE activity between A1 and A2, as well as between A2 and A3, indicating an AChE inhibition. Another significant difference was also observed between A2 and A4.

Metallothionein (MT) content showed slight decrease in mean content measured from A1 to A3 (Fig. 2B). Electronic waste exposure did not have a significant effect on the MT content measured in the test organisms, *F* (3, 39.7) = 0.917, *p* = 0.416, and showed a small effect size of ω^2^ = −0.001.

Superoxide dismutase activity (SOD) indicated a significant effect of e-waste leachate exposure in *D. rerio*, H^2^ (3) = 16.5, *p* < 0.001. The leachate concentrations had a large effect size (ε^2^ = 0.250). Organisms from all leachate treated exposure groups showed SOD activity inhibition compared to A1 (Fig. 2C). The Dunn–Bonferroni Post Hoc analysis revealed significant differences in SOD activity between A1 and A3, as well as between A1 and A4, but not between A1 and A2 or any other group combination.

A significant difference for CAT activity was observed, H^2^ (3) = 14.6, *p* = 0.002, with e-waste leachate exposure having a large effect as signified by the effect size ε^2^ = 0.195. Pairwise comparisons were performed using the Dunn’s procedure with a Bonferroni correction. The Dunn’s test revealed significant differences in CAT activity responses between A1 and A3, as well as between A2 and A3, but no other group combinations (Fig. 2D).

The mean differences for GSH between concentration groups was significant, *F* (3, 27.6) = 3.23, *p* = 0.038; where a Games-Howell Post Hoc analysis revealed that the increase in GSH activity from A1 to A3, was significant and that the e-waste exposure had a large effect size of ω^2^ = 0.099 (Fig. 2E).

For GST activity, a one-way ANOVA indicated a significant difference between groups *F* (3, 71) = 10.8, *p* < 0.001, and a large effect size of ω^2^ = 0.282 (Fig. 2F). The Tukey Post Hoc analysis revealed that the decrease in GST activity from A1 to A3 and A4 were significant. A significant difference between A2 and A3, as well as between A2 and A4 were observed.

A robust Welch ANOVA for MDA content indicated a significant difference within the distribution, *F* (3, 36.2) = 6.68, *p* = 0.001, and a large effect size of ω^2^ = 0.189. A Games-Howell Post Hoc analysis further revealed a significant increase from A1 to A2, as well as, between A2 and A4, and A3 and A4 (Fig. 2G).

Protein carbonyl (PC) content decreased from A1 to A2 and A3, in that order, and showed a slight increase for A4, but the differences between A1 and the exposure concentrations was not significant, *F* (3, 64) = 1.305, *p* = 0.281, and the effect size, ω^2^ = 0.003, indicates a small effect on results obtained (Fig. 2H).

### Energetics

**Fig. 3:**
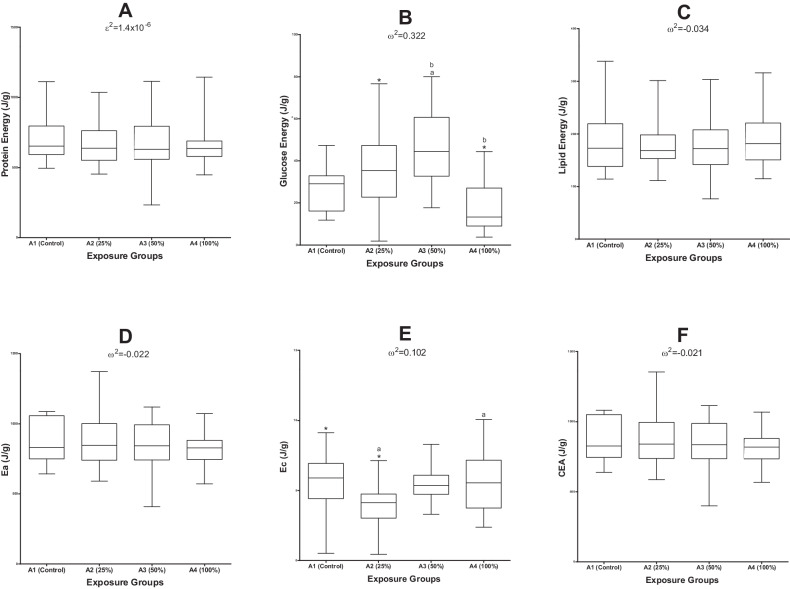
Box-and-whisker plots that show the 5th to 95th percentile range of (**A**)—Protein, (**B**)—Glucose, (**C**)—Lipids, (**D**)—Ea, (**E**)—Ec, (**F**)—CEA. Common superscripts indicate statistically significant differences (p < 0.05) between exposure groups. Omega squared (ω^2^) and epsilon squared (ε^2^) show effect sizes

The overall CEA of the test organisms (Fig. 3) exposed to e-waste leachate, is dependent on Ea (protein, glucose and lipid energy availability) and the Ec by the organisms during the acute exposure study. *Danio rerio* were categorised into four groups based on their exposure concentrations: A1 (n = 20) as the control group, A2 (n = 10) as the 25% leachate exposure group, A3 (n = 10) as the 50% and A4 (n = 10) as the 100% exposure group.

A Kruskal-Wallis H test indicated no significant effect of e-waste leachate exposure on protein energy in *D. rerio*, *H* (3) = 0.736, *p* = 0.865, and the effect size (ε^2^ = 1.4 × 10^−6^) was small. Organisms in the exposed groups had a lower mean protein energy available compared to A1 organisms (Fig. 3A).

The one-way ANOVA showed a significant impact of *F* (3, 75) = 13.5, *p* < 0.001, and a large effect size of ω^2^ = 0.322 for glucose energy available in *D. rerio* (Fig. 3B). The Tukey Post Hoc test revealed that the significance lied between A2, and A4, as well as between A1 and A3 and lastly between A3 and A4.

The standard one-way ANOVA showed no significant differences between exposure groups, *F* (3, 76) = 0.114, *p* = 0.951 and a small effect size of ω^2^ = −0.034, for available lipid energy (Fig. 3C).

The overall Ea results, however, revealed that e-waste leachate exposure did not have any significant effects on Ea in *D. rerio*, *F* (3, 71) = 0.470, *p* = 0.704 (Fig. 3D)

The e-waste leachate exposure imposed a significant effect on Ec, *F* (3, 72) = 3.87, *p* = 0.013. The effect size of e-waste leachate on Ec in *D. rerio* after an acute exposure, was medium (ω^2^ = 0.102). Energy consumption scores decreased for all exposure groups compared to A1. The Tukey Post Hoc test indicated a significant difference between A1 and A2. Similarly, a significant difference was observed between A2 and A4 (Fig. 3E).

A one-way ANOVA was used to assess the overall effects on the energetics of the organisms (Fig. 3F). There was no significant effect of e-waste leachate on CEA in *D. rerio*, *F* (3, 71) = 0.496, *p* = 0.686, and the effect size was small (ω^2^ = −0.021).

### Integrated biomarker response (IBRv2)

**Fig. 4:**
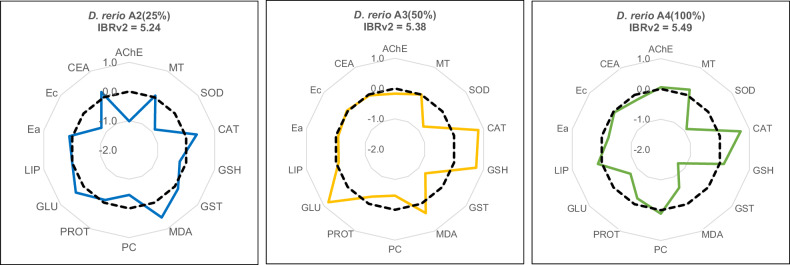
Integrated Biomarker Response values calculated and the associated radar plots for *Danio rerio* after 96 h (acute) exposure to three concentrations of electronic waste leachate

Biomarker responses were assessed with the IBRv2 analysis; which indicated that *D. rerio* showed a greater biomarker response with increasing e-waste leachate concentrations. In A2, AChE activity, SOD activity, GSH content, PC content and Ec had a decreased response to e-waste leachate exposure compared to A1. Catalase activity, GST activity, MDA content, glucose energy, Ea and CEA values were elevated compared to baseline values in A1. There was little to no difference however, in MT, protein and lipid energy. The IBRv2 score for the A2 was 5.24 (Table 5; Fig. 4A2).

**Table 5:** Deviation values and integrated biomarker response scores calculated in *Danio rerio*, for each biomarker, after an acute exposure to electronic waste leachate was concluded

Group	AChE	MT	SOD	CAT	GSH	GST	MDA	PC	Prot	Glu	Lipid	Ea	Ec	CEA	IBRv2
A2 (25%)	1.02	0.063	0.876	0.372	0.224	0.137	0.564	0.461	0.100	0.337	0.001	0.103	0.782	0.199	5.24
A3 (50%)	0.168	0.001	0.804	0.821	0.748	0.719	0.341	0.466	0.262	0.810	0.093	0.076	0.034	0.033	5.38
A4 (100%)	0.055	0.194	0.912	0.703	0.134	1.27	0.604	0.114	0.212	0.719	0.130	0.228	0.036	0.178	5.49

There was a large deviation in CAT activity, GSH content and glucose energy in A3 compared to A1, and MDA content was slightly elevated. The responses for SOD activity, GST activity and PC content were lower compared to the baseline values of A1. The responses measured for MT, Ec, Ea, lipid energy and CEA were relatively similar to A1, with only small deviations. A small decreased response for protein energy and AChE activity was observed. The IBRv2 score for A3 was 5.38, higher than that for A2 (Table 5; Fig. 4**A3**).

The responses measured for SOD activity, GST activity, MDA content and glucose energy available in A4, were notably decreased from baseline values from A1. Similarly, a large increased response was measured for CAT activity. A slightly elevated value for GSH activity, PC content, lipid energy and MT was observed for A4. Small decrease deviations were observed for protein energy, Ea and CEA, whereas little to no deviations were seen for AChE activity and Ec. The IBRv2 score for A4, was 5.49, which is higher than both the A2 and A3 leachate exposure groups (Table 5; Fig. 4**A4**).

## Discussion

The short 'useful' life-expectancy of electronic products—driven by the rapid improvement of innovation, miniaturization and affordability—has led to a major increase in the accumulation of potentially toxic e-waste (Shaikh et al. 2020). This already problematic result is exacerbated by the increase in population, modernization and digitalisation that accompanies the introduction of a fourth IR (Kaya 2019; Opoku and Boachie 2020). Old or used electronics do not die; they pile up in attics or backyards at regular homes or find their way to landfills or ‘e-waste graveyards’ in developing countries (Kaya 2019; Li and Achal 2020). If waste EEE can be leached into the environment, due to moisture, oxidation, rain and improper disposal, it can generate serious environmental problems (Kaya 2019).

Landfills are the most popular method to discard of e-wastes, after materials of value have been extracted, by crude processes such as manual dismantling or burning (Kaya 2019; Shaikh et al. 2020). A vast majority of WPCBs, according to Kaya (2019), were consigned to landfill in 2014, either directly or within the original equipment housing.

Landfills are a popular waste treatment method, with a long history and wide applicability. They are a simple and cost-effective operation to discard of unwanted and unregulated waste (Zhou et al. 2019). Landfills represent a non-sustainable loss of finite materials and are adding to the burden of increasing environmental health concerns, through surface runoff and precipitation infiltration (Opoku and Boachie 2020; Przydatek and Kanownik 2021). Typically, the leached residues are also discharged directly into rivers or are drained into tailing dams, which introduce the toxic compounds directly into aquatic ecosystems and threaten the health of the environment and humans alike (Zhou et al. 2013; Zhou et al. 2019).

### Electronic waste leaching

Electronic waste and WPCBs are described as being very complex materials, and the exact composition analysis thereof, is difficult to determine, due to the heterogeneous composite nature of the materials (Kaya 2019). The complex composition of small components associated with e-waste and WPCBs results in different chemical compositions between different e-waste leachate batches (Xiu et al. 2015; Borthakur 2020). Waste printed circuit boards are considered an appropriate component of e-waste to assess metals leached because they are referred to as the ‘urban mines’ of e-waste. They are resource rich and house 40% of the metals, 30% of the organics and 30% of the ceramics that make up or are associated with e-waste (Mmereki et al. 2016; Rao et al. 2017; Zeng et al. 2020). There is however, great variance in composition of WPCBs originating from different appliances manufacturers and production year, resulting in different conclusions on the exact composition of metals found in e-waste (Kaya 2019; Ghimire and Ariya 2020).

Extractive metallurgy is a field in metallurgical engineering dedicated to the separation of metals from minerals and materials that may contain them (Torres et al. 2018). Since the emergence of e-waste, extractive metallurgy has been extensively employed to aid in the leaching and extraction of valuable metals contained within the discarded e-waste (Torres et al. 2018; Barragan et al. 2021). Many published studies on metal recovery from e-waste, typically make use of hydrometallurgical processes that depend on the use of aqueous solutions to extract metals, at low temperatures and cost-effective implementation, which makes this process a favourable and practical option (Barragan et al. 2021). Acids, such as H_2_SO_4_, HCl and HNO_3_, are popularly used as leaching mediums because of their ‘fast leaching kinetics’ and their ability to form stable complexes with selective base metals, leached from the wastes (Barragan et al. 2021). Pure acidic mediums used to leach waste’ metals have proven to be effective—its efficiency when diluted with water to simulate natural environmental leaching, however, has to be assessed (Torres et al. 2018).

The leaching procedure for this study was completed by following the non-acidic dynamic test leaching’s procedure, where the dismantled e-waste components were submerged in borehole water, in a ratio of 1:10 (Solid: Liquid) and contained submerged air-stones to simulate water flow over the components. The final leaching cycle took place in a controlled environmental room, with a constant temperature of 28 °C. The elemental bioavailability was only assessed after the four-week leaching cycle was concluded and it was found that from the twenty-five metals tested for with ICP-MS, only 19 were within detectable ranges. A graphical representation of the tank and the metals highest in concentration is presented as percentages, in Fig. 1; which indicates the metal distribution with Ni > Ba > Li > Zn > Fe > Al > Cu as the most abundant metals in concentration.

A study by Zhou et al. (2019), found that metals with a higher atomic mass dissolved faster, compared to metals with a lower atomic mass and that the concentration of these metals were higher in the leaching medium, as the particle size of the waste components decreased. The smaller the particle size, the larger the surface area for the medium to react with, and the faster the dissolution of metals (Zhou et al. 2019; Meem et al. 2020). The WPCBs used in this study were manually dismantled and cut up into smaller pieces of varying sizes, whereas in most e-waste composition related research the e-waste PCBS would be milled before being assessed for the metal composition leached. This is done to ensure that the surface area of all components can be considered identical, and a more accurate depiction of metals leached can be seen (Pant et al. 2012; Petter et al. 2014; Zhou et al. 2019).

The metals highest in bioavailability, namely: Ni, Ba, Zn, Li, Fe, Al and Cu are all classified as common base metals elements that oxidise and corrode easily (Pant et al. 2012). According to Torres et al. (2018), effective metal leaching is dependent on several factors, such as the concentration of the leaching medium (pH), the temperature, and the solubility of the reaction products – in this case, the e-waste WPCBs.

For leaching to occur, the metals must be oxidised to its ionic state, to have a higher degree of solubility, which may take time, unless the temperature or pH of the medium in which the leaching occurs in, is greatly adjusted (Torres et al. 2018). The final leaching cycle was conducted over a four-week period, and although the metals did start to oxidise and corrode. An investigation into a longer natural leaching process is encouraged. E-waste leachate surface-runoff into the ambient environment from landfills happens over a longer span of time (Zhou et al. 2019). Therefore, the toxicity of the metals leached from e-waste may increase with time.

### Acute exposure

The exposures of *D. rerio* took place under controlled environmental conditions to ensure and maintain stable conditions throughout the exposure periods and limit the effects different temperature fluctuations could induce on the organisms’ stress responses.

Some xenobiotic substances can result in physiological and biochemical alterations in organisms, even if the substance is not bioavailable. These changes can occur in many forms, as enzyme activity can either be stimulated or inhibited by the substance, depending on the physical properties of the xenobiotic substance, as well as the type of enzyme and the function it serves (van der Oost et al. 2003; Pikula et al. 2019). Reactive oxygen species (ROS) are naturally occurring cytotoxic molecules, which increase when harmful substances enter the cells of living organisms. Fish, exposed to xenobiotic substances, therefore, produce antioxidant defences to neutralise these excess harmful ROS, and thereby try to limit oxidative stress. Specific macromolecules, namely proteins and lipids, can be damaged by their reactions with a xenobiotic substance, as the presence of ROS increase (Marnett 1999; Wehr and Levine 2013). Therefore, the physiological and biochemical changes that occurred in *D. rerio* following the exposure to e-waste leachate, was assessed with biomarkers of effect and biomarkers of exposure.

A wide range of toxins and compounds can affect AChE activity, and the enzyme activity can either be stimulated or inhibited. Metal exposure has been shown to have inhibitory effects on AChE activity for fish (Xuereb et al. 2009). The exposure of *D. rerio* to the leachate did not show a consistent trend during the exposure for AChE activity. An inhibitory effect for the lower exposure concentration was observed, compared to a stimulatory effect for A4—but there was also less of an inhibition for A3, compared to A2 observed. Some studies suggest that metals such as Cu could cause a stimulatory effect on AChE activity, but Pb, Cd and Zn could all have an inhibitory effect rather than a stimulatory effect (Araújo et al. 2018; Gerber et al. 2018; Watson et al. 2021).

Metalloproteins play a vital role in trace-metal homoeostasis and metal detoxification; MT content can be initiated through numerous other mechanisms, such as the increase in ROS, and is not necessarily linked to metal exposure only (Viarengo et al. 1997; Wang et al. 2012; Kroon et al. 2017). The antioxidant defence mechanisms can easily be overwhelmed if MT detoxification is not efficient enough in removing bioavailable metals in the organism. This typically leads to irreversible damage to essential macromolecules, as the metals bind to macromolecules, such as proteins and lipids (Wang et al. 2012). No significant differences were found between A1 and leachate exposure group for MT content.

Electronic waste exposure did, however, have a significant effect on SOD activity measured in *D. rerio*; organisms in the exposure groups with the highest concentration of e-waste leachate, measured with the lowest SOD activity compared to the lower concentration exposures and group A1. A decrease in mean SOD activity is therefore seen from group A1 to A4. Superoxide dismutase activity is an enzyme that functions as a reducing agent for an increase in ROS. Metal exposure is expected to result in a stimulatory effect in SOD activity. However, one study showed that an increase in exposure concentration to some environmental contaminants may induce an inhibitory SOD response (Pedrajas et al. 1995). The O_2_^-^ reactive oxygen radical could also have potentially reacted with the surrounding molecules in solution, to generate OH^-^, and that would also have limited the SOD response needed (Abou-Seif 1998). At low concentrations, the exposure to e-waste leachate may have induced an increase in SOD activity as ROS were generated, but as the concentration of leachate increased, SOD may have been inhibited, either because of molecules reacting or contaminants interfering with the antioxidant defence mechanism of the organism.

The catalase enzyme functions as the second line of defence to the increase in ROS – it reduces H_2_O_2_ produced by SOD, to O_2_ and H_2_O (van der Oost et al. 2003). The acute exposure analysis on *D. rerio* showed a significant effect of e-waste leachate exposure on CAT activity between group A1 and the two lower concentration groups, but not to the A4 group. Metals such as Cd, Cr and Zn have been shown to cause significant increases in CAT responses in *Oreochromis niloticus* (Atli et al. 2006). The increase in CAT activity can be attributed to an increase in oxidative stress caused by exposure to metals in the e-waste leachate (Pikula et al. 2019).

Reduced glutathione enzyme, acts as a free radical scavenger and has been identified as an important defence mechanism to oxidative stress (Winterbourn and Metodiewa 1994; Chavan et al. 2005). An increase in metal exposure has been found to correlate with an increase in GSH levels (Viarengo et al. 1997). The GSH levels measured between the groups was not significant, although the A4 group presented the highest measured GSH response. Reduced glutathione and MTs are both cysteine rich molecules. So, there might be a competition between these molecules binding to metals and resulting in either one of the two being stimulated, and the other one inhibited in activity (Giralt et al. 1993).

Glutathione S-transferase on the other hand, is the enzyme that aids the conjugation of GSH to free radicals in the organisms’ body, and therefore, works in conjunction with GSH to limit the accumulation of a xenobiotic substance in organisms exposed to a potential toxicant (Tsuchida 2002). Electronic waste leachate exposure had a significant effect on GST activity in the acute exposure of *D. rerio*, where a decrease in mean GST measured was observed from A1 to the highest leachate exposure group—GST inhibition occurred as the exposure concentration increased. According to Özaslan et al. (2017), certain metals could function as GST inhibitors, such as Zn, Cd, Pb and Hg.

Exposure to e-waste leachate showed a significant measured effect for the MDA content on *D. rerio*. An increase in MDA content for the A2 and A3 groups compared to A1 group was seen, but also a decreased MDA content for A4 group. Studies suggest lipid peroxidation to be one of the most considerable impacts observed for metal exposure (van der Oost et al. 2003; Üner et al. 2006; Garcia et al. 2020). Severe lipid peroxidation could lead to deleterious effects in the organisms’ health – as lipid components of cellular structures are damaged and apoptosis occurs (Üner et al. 2006). According to Garcia et al. (2020), fish are capable of excreting MDA, which could be why an inhibition in MDA content for the highest exposure concentration group was seen compared to the lower concentration exposure groups. A stimulatory MDA response to As, Cd, Cu, Pb and Zn exposure in the African catfish, *Clarias gariepinus*, was observed in a study by Farombi et al. (2007).

Protein carbonyl content is referred to as a standard indicator of protein macromolecular damage caused by the increase in ROS (Wehr and Levine 2013). No significant effect of e-waste leachate exposure on PC content was seen, but an inhibition of PC content for the lower exposure concentration groups compared to a stimulatory PC content for the highest leachate exposure group was noted in the IBRv2 results. Metals typically have a greater affinity for lipid macromolecules than for protein, so a more pronounced physiological effect on fatty tissue, compared to protein structures is expected (Farid et al. 2012; Ardeshir et al. 2017).

### Energetic following the acute exposure

Cellular energy allocation is used as an indicator of an organism’s total energy when exposed to a potential environmental contaminant. Any stress response can be induced by the exposure to a pollutant, which would result in metabolic changes (Wang et al. 2012). The exposure to any pollutant may result in an increased metabolic response, as an attempt to mitigate the harmful effects the contaminant can induce. Therefore, total energy analysis is investigated by looking at the energy availability (Ea) for metabolic function and total energy expenditure (Ec) or energy consumption of the organism (De Coen and Janssen 2003; Fedorova et al. 2014; Martini et al. 2015).

The Ea in an organism is composed of the energetic equivalents of protein, glucose, and lipids (De Coen and Janssen 2003). It is expected that e-waste exposure would result in decreased protein energy measured, as these macromolecules can be denatured by an increase in ROS induced by metal exposure (Fedorova et al. 2014). No significant impact on protein energy between the A1 group and the leachate exposure groups were observed, and little deviation in group means were observed. Similarly, an increase in ROS typically results in lipid peroxidation, and a decrease in lipid energy between the exposure groups is expected, but again, no significant difference between the groups were found (Roess et al. 2008). A relationship between lipid peroxidation and lipid energy available can be seen however, where the MDA content decreases with increasing exposure concentration, but a decrease in lipid energy available is seen as the concentrations increase. Deviations in group means are observed when assessing the glucose energy. An increase in glucose energy is observed for the lower leachate concentration groups compared to the A1 group, but a decrease is observed for the A4 group. This difference was not significant. The acute e-waste leachate exposure on *D. rerio*, therefore revealed no significant effect on the overall Ea between the A1 group and exposure groups.

When assessing the energy expenditure for *D. rerio* exposed to e-waste leachate, it was found that the differences between groups were significant. The organisms showed little increase for the two highest leachate concentration groups, compared to group A1. The A2 group, however, showed a significant decreased deviation from the means measured for the other groups. So, a decreased metabolic activity for the lowest e-waste concentration group was observed.

Similar group readings for overall CEA were observed for this acute exposure on *D. rerio*. The highest leachate concentration group (A4), measured with a slightly lower mean CEA than the other groups, but the difference was not significant. This result can be explained by the slightly lowered Ea, along with a slightly higher Ec found for organisms in the A4 group.

The IBRv2 was conducted to indicate the multi-biomarker responses in *D. rerio* exposed to e-waste leachate. These IBRv2 scores showed that there was a greater biomarker response, for the acute study on *D. rerio* as the exposure concentration increased (Fig. 4). From the radar plots for the IBRv2, it can be seen that as the concentration increased, a stimulated response in AChE activity for A4, compared to the inhibited AChE response for A2 group, and slightly less inhibited response for the A3 group, resulted. A similar trend for the CAT, PC, lipid energy and Ec biomarkers, were observed where, as the exposure concentration increased, an increase in biomarker response was also seen. The biomarker response of SOD, GST, MDA, Ea, protein energy and CEA showed the opposite trend, where an inhibition in the measured responses was observed as the exposure concentration increased. The MT biomarker showed no significant deviation from the baseline values of the A1 group. Biomarkers such as GSH and glucose energy showed both inhibition and stimulation. Reduced glutathione illustrated an inhibited IBR response for the A2 group, but stimulatory responses for the A3 and A4 groups, and the glucose energy has stimulatory responses for the lower exposure concentrations, and an inhibitory response for A4 group.

## Conclusion

This study aimed to determine the broad array of metal elements typically associated with e-waste leachate. This aim was achieved by leaching WPCBs; where the non-acidic dynamic leaching procedure was applied, because it mimics natural environmental leaching. The metals found highest in concentration during the leaching processes were Ni, Ba, Li, Zn, Fe, Al and Cu, in that order. Twenty-five different metal elements present in the simulated e-waste leachate were expected, however, only nineteen elements were within detectable ranges on the ICP-MS. The nineteen metals, in ascending order of concentration, were as follows:

Y < Au < Cr < Ge < As < Ag < Mo < Co < Pb < Sn < Ga < Sb < Cu < Al < Fe < Zn < Li < Ba < Ni

The second aim of this study was to assess the combined synergistic, antagonistic or additive effect of these metals on *D. rerio*, following an acute exposure study of 96-hrs to simulated e-waste leachate. It was hypothesized that the acute exposure of *D. rerio* to simulated e-waste leachate would result in measurable biochemical and physiological responses. These responses were assessed with biomarkers of exposure and effect. Exposure to e-waste leachate resulted in significant effects measured for AChE, SOD, CAT, GSH, GST, MDA, glucose energy, and Ec. Therefore, the hypothesis was supported.

The results obtained in this study, therefore, indicate that the combined effects of metals leached from e-waste WPCBs induced physiological and biochemical alterations in *D. rerio* at considerably low metal concentrations. It was also hypothesised that the increase in exposure concentrations will positively correlate to an increase in measurable biomarker responses; this hypothesis was supported by the increase in IBRv2 score obtained. The IBRv2 score increased as the exposure concentration increased, indicating the additive effect of increased metal concentration exposure induced during a 96 h exposure study.

## Supplementary information

Supplementary Table

## Data Availability

Data is provided within the manuscript or supplementary information. A full unpublished copy of the MSc thesis is available at https://ujcontent.uj.ac.za/esploro/outputs/graduate/Biomarker-responses-in-Danio-rerio-following/9927007707691?institution=27UOJ_INST.
