# Supplementary material for: Biomarker responses in Danio rerio following an acute exposure (96 h) to e-waste leachate
Source: Ecotoxicology. 2024 Jul 12;33(8):859–74. doi: 10.1007/s10646-024-02784-6 (PMC11399175; doi:10.1007/s10646-024-02784-6)
Supplement: Supplementary file 1 — Supplementary Table [file 10646_2024_2784_MOESM1_ESM.docx]

Supplementary Table: Fish dissection data sheet indicating weight and sex of the fish used for biomarker analysis.

| Conc. | Code | Fish | Standard Length (cm) | Total Length (cm) | Total weight (g) | Sex | Vicera weight (g) | Left Muscle (g) | Right Muscle (g) |
| --- | --- | --- | --- | --- | --- | --- | --- | --- | --- |
| 0% | 1A01 | 1 | 34,80 | 49,12 | 0,7898 | F | 0,1208 | 0,0732 | 0,0779 |
|  | 1A02 | 2 | 34,47 | 48,17 | 0,7824 | F | 0,0752 | 0,1002 | 0,1191 |
|  | 1A03 | 3 | 34,99 | 50,45 | 0,9289 | F | 0,2122 | 0,0867 | 0,1124 |
|  | 1A04 | 4 | 36,51 | 52,78 | 0,9217 | F | 0,1356 | 0,1639 | 0,1343 |
|  | 1A05 | 5 | 38,85 | 52,33 | 0,7933 | F | 0,1078 | 0,0778 | 0,0488 |
|  | 1A06 | 6 | 35,91 | 51,16 | 0,8268 | F | 0,1644 | 0,0734 | 0,0970 |
|  | 1A07 | 7 | 39,33 | 54,44 | 0,9435 | F | 0,1200 | 0,1122 | 0,0963 |
|  | 1A08 | 8 | 34,87 | 50,55 | 0,7932 | F | 0,1283 | 0,1322 | 0,0708 |
|  | 1A09 | 9 | 35,93 | 48,78 | 0,9358 | F | 0,1670 | 0,1161 | 0,0975 |
|  | 1A10 | 10 | 36,20 | 51,37 | 0,8026 | F | 0,1348 | 0,941 | 0,0986 |
| 0% | 2A01 | 1 | 38,11 | 54,1 | 0,8282 | F | 0,1316 | 0,0782 | 0,0713 |
|  | 2A02 | 2 | 37,17 | 51,32 | 0,9304 | F | 0,1447 | 0,0987 | 0,0755 |
|  | 2A03 | 3 | 35,53 | 51,02 | 0,869 | F | 0,1474 | 0,0801 | 0,0732 |
|  | 2A04 | 4 | 35,44 | 48,83 | 0,8311 | F | 0,1389 | 0,1034 | 0,1284 |
|  | 2A05 | 5 | 38,35 | 51,41 | 0,9967 | F | 0,1809 | 0,0925 | 0,1088 |
|  | 2A06 | 6 | 36,91 | 50,92 | 0,8725 | F | 0,1426 | 0,1312 | 0,1123 |
|  | 2A07 | 7 | 35,79 | 54,19 | 0,8386 | F | 0,2659 | 0,0759 | 0,093 |
|  | 2A08 | 8 | 39,21 | 53,51 | 0,9664 | F | 0,1733 | 0,1433 | 0,1516 |
|  | 2A09 | 9 | 36,79 | 50,87 | 0,8442 | F | 0,1277 | 0,0952 | 0,0787 |
|  | 2A10 | 10 | 40,44 | 53,42 | 0,8792 | F | 0,1178 | 0,1069 | 0,1431 |
| 25% | 1B01 | 1 | 40,60 | 52,14 | 0,8711 | F | 0,1508 | 0,1089 | 0,0996 |
|  | 1B02 | 2 | 39,43 | 52,47 | 0,8170 | F | 0,1152 | 0,1129 | 0,1059 |
|  | 1B03 | 3 | 37,48 | 48,53 | 0,8457 | F | 0,1403 | 0,0818 | 0,0867 |
|  | 1B04 | 4 | 40,98 | 53,99 | 0,9350 | F | 0,1653 | 0,1340 | 0,1243 |
|  | 1B05 | 5 | 50,51 | 54,20 | 0,7474 | M | 0,0282 | 0,1006 | 0,0877 |
|  | 1B06 | 6 | 39,77 | 50,39 | 0,8902 | F | 0,1519 | 0,0916 | 0,1230 |
|  | 1B07 | 7 | 40,58 | 49,73 | 0,8183 | F | 0,1554 | 0,0647 | 0,0797 |
|  | 1B08 | 8 | 40,28 | 52,50 | 0,8211 | F | 0,0920 | 0,1444 | 0,1245 |
|  | 1B09 | 9 | 39,48 | 50,44 | 0,8483 | F | 0,1278 | 0,0867 | 0,0890 |
|  | 1B10 | 10 | 39,80 | 54,07 | 0,8809 | F | 0,1608 | 0,1263 | 0,1093 |
| 25% | 2B01 | 1 | 37,50 | 46,50 | 0,8114 | F | 0,1625 | 0,102 | 0,0821 |
|  | 2B02 | 2 | 37,00 | 49,50 | 0,8010 | F | 0,0611 | 0,0751 | 0,1091 |
|  | 2B03 | 3 | 36,80 | 48,60 | 0,7550 | F | 0,0331 | 0,0221 | 0,0571 |
|  | 2B04 | 4 | 36,90 | 50,10 | 0,6510 | M | 0,0414 | 0,0691 | 0,0530 |
|  | 2B05 | 5 | 36,00 | 44,50 | 0,5931 | M | 0,0321 | 0,1002 | 0,1422 |
|  | 2B06 | 6 | 36,40 | 48,60 | 0,8251 | M | 0,0871 | 0,0411 | 0,0681 |
|  | 2B07 | 7 | 36,00 | 51,00 | 0,8551 | F | 0,1296 | 0,0481 | 0,0936 |
|  | 2B08 | 8 | 37,70 | 50,50 | 0,8190 | F | 0,1221 | 0,1203 | 0,1002 |
|  | 2B09 | 9 | 35,90 | 48,30 | 0,8761 | F | 0,1197 | 0,7696 | 0,0890 |
|  | 2B10 | 10 | 36,10 | 50,30 | 0,6741 | F | 0,1004 | 0,0714 | 0,0771 |
| 50% | 1C01 | 1 | 36,89 | 52,47 | 0,7793 | F | 0,1042 | 0,0736 | 0,0913 |
|  | 1C02 | 2 | 38,56 | 51,26 | 0,9983 | F | 0,1836 | 0,0785 | 0,1950 |
|  | 1C03 | 3 | 36,36 | 51,43 | 0,9042 | F | 0,1539 | 0,0829 | 0,0790 |
|  | 1C04 | 4 | 38,52 | 52,15 | 0,8165 | F | 0,1074 | 0,0842 | 0,1079 |
|  | 1C05 | 5 | 39,03 | 52,60 | 0,8392 | F | 0,1062 | 0,1116 | 0,0655 |
|  | 1C06 | 6 | 37,77 | 50,37 | 0,7699 | F | 0,1010 | 0,0858 | 0,0992 |
|  | 1C07 | 7 | 37,97 | 53,49 | 0,8476 | F | 0,1383 | 0,0804 | 0,1205 |
|  | 1C08 | 8 | 35,75 | 47,09 | 0,7496 | M | 0,0465 | 0,0901 | 0,1155 |
|  | 1C09 | 9 | 36,27 | 48,51 | 0,7534 | F | 0,1044 | 0,0607 | 0,2469 |
|  | 1C10 | 10 | 37,46 | 49,86 | 0,9092 | F | 0,1751 | 0,1117 | 0,1196 |
| 50% | 2C01 | 1 | 36,56 | 51,46 | 0,9303 | F | 0,1303 | 0,0980 | 0,0874 |
|  | 2C02 | 2 | 36,49 | 51,46 | 0,6842 | F | 0,0926 | 0,0997 | 0,0987 |
|  | 2C03 | 3 | 39,93 | 53,75 | 0,9260 | F | 0,1353 | 0,0599 | 0,0695 |
|  | 2C04 | 4 | 37,95 | 48,09 | 0,8588 | F | 0,1108 | 0,1229 | 0,1295 |
|  | 2C05 | 5 | 35,90 | 52,75 | 0,5000 | F | 0,1316 | 0,0936 | 0,0743 |
|  | 2C06 | 6 | 38,60 | 55,21 | 0,9009 | F | 0,1436 | 0,1127 | 0,1009 |
|  | 2C07 | 7 | 38,24 | 48,39 | 0,8068 | F | 0,1379 | 0,0763 | 0,0549 |
|  | 2C08 | 8 | 35,63 | 50,10 | 0,7880 | F | 0,1445 | 0,1077 | 0,0926 |
|  | 2C09 | 9 | 36,38 | 48,74 | 0,7433 | F | 0,1001 | 0,0536 | 0,0590 |
|  | 2C10 | 10 | 37,59 | 50,84 | 0,7863 | F | 0,1026 | 0,0859 | 0,0971 |
| 100% | 1D01 | 1 | 36,40 | 51,80 | 0,7645 | F | 0,1096 | 0,0541 | 0,0486 |
|  | 1D02 | 2 | 35,62 | 48,50 | 0,7138 | F | 0,0865 | 0,1017 | 0,0981 |
|  | 1D03 | 3 | 36,13 | 52,61 | 0,7502 | F | 0,1056 | 0,0747 | 0,0885 |
|  | 1D04 | 4 | 38,89 | 55,22 | 0,8253 | F | 0,1111 | 0,1312 | 0,1244 |
|  | 1D05 | 5 | 36,64 | 50,69 | 0,8756 | F | 0,1844 | 0,0872 | 0,0688 |
|  | 1D06 | 6 | 36,12 | 47,95 | 0,7940 | F | 0,1478 | 0,0853 | 0,1042 |
|  | 1D07 | 7 | 37,41 | 54,55 | 0,8452 | F | 0,1339 | 0,0568 | 0,1061 |
|  | 1D08 | 8 | 35,09 | 49,97 | 0,7739 | F | 0,1204 | 0,1180 | 0,1205 |
|  | 1D09 | 9 | 36,20 | 52,16 | 0,7090 | F | 0,1014 | 0,0849 | 0,0602 |
|  | 1D10 | 10 | 36,49 | 51,15 | 0,8819 | F | 0,1132 | 0,1148 | 0,1330 |
| 100% | 2D01 | 1 | 37,87 | 52,84 | 0,8042 | F | 0,1168 | 0,0884 | 0,0813 |
|  | 2D02 | 2 | 36,32 | 50,33 | 0,7667 | F | 0,1438 | 0,0925 | 0,1004 |
|  | 2D03 | 3 | 34,31 | 49,10 | 0,8845 | F | 0,1473 | 0,0856 | 0,1050 |
|  | 2D04 | 4 | 36,13 | 50,78 | 0,7772 | F | 0,1108 | 0,1249 | 0,0876 |
|  | 2D05 | 5 | 37,90 | 53,10 | 0,9121 | F | 0,1678 | 0,1127 | 0,0932 |
|  | 2D06 | 6 | 38,15 | 53,80 | 0,8768 | F | 0,1291 | 0,1463 | 0,1376 |
|  | 2D07 | 7 | 36,15 | 50,43 | 0,7172 | F | 0,1119 | 0,0937 | 0,0630 |
|  | 2D08 | 8 | 34,90 | 50,81 | 0,8033 | F | 0,1362 | 0,1232 | 0,1134 |
|  | 2D09 | 9 | 34,64 | 49,36 | 0,7728 | F | 0,1373 | 0,0649 | 0,0713 |
|  | 2D10 | 10 | 34,77 | 50,05 | 0,7360 | F | 0,0917 | 0,1030 | 0,0976 |
